# Reframing data ethics in research methods education: a pathway to critical data literacy

**DOI:** 10.1186/s41239-023-00380-y

**Published:** 2023-02-20

**Authors:** Javiera Atenas, Leo Havemann, Cristian Timmermann

**Affiliations:** 1 Centre for Excellence in Learning and Teaching, University of Suffolk, Ipswich, UK; 2 Arena Centre for Research-Based Education, University College London, London, UK; 3 Institute of Educational Technology, The Open University, Milton Keynes, UK; 4 Ethics of Medicine, Medical Faculty, University of Augsburg, Augsburg, Germany

**Keywords:** Critical data literacy, Data literacy, Ethics, Data ethics, Research methods, Curriculum design, Higher education

## Abstract

This paper presents an ethical framework designed to support the development of critical data literacy for research methods courses and data training programmes in higher education. The framework we present draws upon our reviews of literature, course syllabi and existing frameworks on data ethics. For this research we reviewed 250 research methods syllabi from across the disciplines, as well as 80 syllabi from data science programmes to understand how or if data ethics was taught. We also reviewed 12 data ethics frameworks drawn from different sectors. Finally, we reviewed an extensive and diverse body of literature about data practices, research ethics, data ethics and critical data literacy, in order to develop a transversal model that can be adopted across higher education. To promote and support ethical approaches to the collection and use of data, ethics training must go beyond securing informed consent to enable a critical understanding of the techno-centric environment and the intersecting hierarchies of power embedded in technology and data. By fostering ethics as a method, educators can enable research that protects vulnerable groups and empower communities.

## Introduction

Data permeates every dimension of our lives, as numbers are used to rate, compare, and allocate us into different categories. Our own data is used to define our worth, measure our effectiveness and, in a myriad of other ways, to inform or construct what we are today. We are ‘governed by numbers’—subject to numbers and numbered subjects (Ball, 2015; Ozga, 2008), so are our scholarly practices, including teaching and research (Grant, 2022).This situation has been widely touted as a breaking dawn of increasingly information based on ever-expanding volumes of data, critical voices have observed that everyone and everything is now quantified and subject to automated and potentially discriminatory decision-making processes (Eubanks, 2018; Kleinberg et al., 2018; Lambrecht & Tucker, 2019). Research education must acknowledge that datafication of society has an impact on how research is conducted and the ethical challenges researchers and data scientists face in order to conduct studies that benefit society.

In this datafied environment, higher education (HE) educators and students must become aware of how technology driven data collection and processing affects themselves and others, and develop a critical research approach to the use of data, including technical and socially driven data literacies (Atenas et al., 2020; Ross et al, 2022; Williamson et al., 2020). Current educational disadvantages in relation to critical data literacies risk rendering people into mere objects of both history (Freire, 1968) and data (Johnson, 2014), exacerbating existing inequalities. Indeed, according to the European Union’s recent framework for digital competence,

Everyone should acquire a basic understanding of new and emerging technologies including AI. This has the potential to support them to engage positively, critically and safely with this technology, and be aware of potential issues related to ethics, environmental sustainability, data protection and privacy, children rights, discrimination and bias, including gender bias and disability and ethnic and racial discrimination. (Redecker & Punie, 2020, p.14)

Such requirements point to the need for pedagogies that foster the development of lifelong and lifewide learning and transversal skills across the disciplines. In particular, it is incumbent upon those designing research methods, data science and data literacy programmes and learning units to systematically incorporate critical, ethical and political dimensions of data and datafication.

The critical orientation of our approach arises from the need, as identified in critical theory (Bohman, 2005; Bronner, 2009; Foucault, 1980) and in education, critical pedagogy (Freire, 1968; Giroux, 2010), to challenge economic and political domination by fostering inquiry into the operations and structures of power. Thus, we seek to support the development of up-to-date research methods courses that develop skills not just in technical data management and analysis, but support the development of critical data literacy and of the concept of agency in students as future researchers, so they are aware of and acknowledge the circumstances of oppression in the current pervasive datafication of human beings and society, often in the service of surveillance capitalism (Zuboff, 2015).

Therefore, in this paper we adopt an approach aligned with critical data literacy, defined by Brand and Sander (2020) as “the ability to critically engage with datafication by reflecting on the societal implications of data processing and implementing this understanding in practice” (p. 2). Drawing upon the aforementioned broad, critical orientations to society and education, we also situate ourselves in conversation with an emerging literature of critical data studies which is especially concerned with challenging power dynamics in the context of datafication (e.g. Hepp et al., 2022; Iliadis & Russo, 2016; Markham, 2018; Pangrazio & Selwyn, 2019; Richterich, 2018; Tygel & Kirsch, 2016). This article supports a re-framing of research methods and to ‘zoom in’ on the specific role of *data ethics* in data literacy and the shaping of research practices according to the degree to which the ethical dimensions of data are understood and considered.

Ethics is generally concerned with the analysis of what is good for society and individuals. Applications of ethics to fields as diverse as medicine and environmental protection are now well-established, and data ethics is developing into a distinct branch of applied ethics (Véliz, forthcoming). In this article we follow a broad definition of data ethics, as the study and evaluation of moral problems arising from data (including its uses and harvesting), algorithms and corresponding practices, in view of developing morally desirable solutions (Floridi & Taddeo, 2016). According to this understanding, data ethics works at a higher level of abstraction than information ethics, as information ethics deals with practices where data is already given a meaning and an interpretation (Floridi, 2010; Floridi & Taddeo, 2016). It should also be understood as a core element of data literacy; for Prado and Marzal (2013), data literacy is a skillset that “enables individuals to access, interpret, critically assess, manage, handle and ethically use data” (p. 126).

While we contend that the need for improved data literacy and data ethics is universal (Atenas et al., 2020), we focus particularly on the role research methods educators can play, as they foster the development of future researchers, and therefore, are uniquely positioned to address this need. To effectively implement action-guiding ethical principles, we adopt an ethics of care perspective as complementary to our critical orientation to data, as it examines how data affects social and political relationships, rather than only individual interests (Held, 2006; Noddings, 1988, 2017; Robinson, 2011; Tronto, 1993). It seems clear that there is a need for a holistic and inclusive approach to the embedding of data ethics training, but it is unclear to which extent such concerns are present in the existing curriculum, or whether they are discussed at all. In designing this study we therefore focused our efforts on answering the following research questions:

1. To what extent is data ethics currently incorporated and represented in
  - existing academic training in research methods and data science
  - data ethics frameworks from diverse sectors? And,
2. According to relevant, and especially critically-oriented literature, what are the key and emerging areas of ethical concern in working with data, that should therefore be included into programmes of study covering research methods and data literacies?

To promote ethically-informed critical data literacy programmes in taught courses in HE, we have assembled a data ethics framework based upon a three-part review which examined data ethics issues from a range of areas including research and data science, industry, government, education, public, private and civil society sectors, and academic literature.

The purpose of this paper is to both establish the need for, and outline, a set of action-guiding ethical principles for embedding data ethics as a core element in teaching data skills within research methods and data science training, in which ethics is understood not just as a learning unit, but a core transversal component across the elements of the research cycle, from data collection to analysis. Understanding the ethical conundrums inherent in working with data is core to the challenge of considering how data collected for an initial purpose might be put to other uses in the future, and how data from different sources may be combined into new datasets that can remove anonymity or be used to predict and influence behaviour (Hand, 2018). The ascendancy of techno-solutionism amid a fluid technological landscape makes the precise shape of future data threats difficult to discern; but experience suggests that the already marginalised and vulnerable carry the most risks. Sustainable ethical principles to guide decision-making in data practices, in education and beyond, are urgently needed.

## Methods

To identify the different ethical concepts and approaches under discussion we examined three sources dealing with data uses and harvesting: syllabi in higher education courses, ethical frameworks and academic literature.

### Identification and selection of syllabi

We identified the different syllabi through a non-systematic internet search using Google with the keywords “data ethics”, “digital ethics” or “data justice” in combination with “course” and “university”. We repeated this search with the corresponding terms in Spanish, Portuguese, Italian, French and German, to complement this study with the language competence of the authors.

First, we searched for the term “ethics” in research methods curricula, initially retrieving over 600 records. Identified records were reviewed to determine if a description of the course units and content was provided, on the first pass narrowing the sample to 340 records which provide some information. On further investigation, only 250 of these provided sufficient detail to extract relevant information about how (or if) data ethics is taught at undergraduate (118), master’s (81) and doctoral level (51). Second, we aimed to identify postgraduate-level data science programmes with prospectuses available to download or review online. This yielded 170 programmes, of which 80 provided a detailed description of the modules, including the units taught, so we could identify how or if data ethics was embedded in curricula. To ensure a minimum quality level, we only included syllabi from universities. Syllabi that were not available online or only offered a brief synopsis were excluded.

In total, we reviewed the inclusion of data ethics in research methods courses from 250 syllabi (quantitative, qualitative and hybrid in focus) and 80 postgraduate programmes in data science, which were taught between 2017 and 2021 in the US, UK, Spain, France, Germany, Brazil, Portugal and Italy.

### Identification and selection of ethical frameworks

In a second step, we reviewed a series of existing frameworks on data ethics, drawn from academia, public sector organisations, civil society, and industry. We searched for academic literature and web-based information on structured frameworks, which contain underpinning ethical principles, pillars or skills in order to identify which elements were discussed and how these were described by the different organisations promoting data ethics. To identify these frameworks, we carried out a non-systematic literature search using Google and DuckDuckGo with the keywords: “ethics” and “data” along with “framework”, “guideline”, “principle” or “recommendation”. The use of two databases was intended to reduce algorithmic biases within each of these databases and avoid missing important sources. As quality control criteria, we only included frameworks from institutions with international reputation. We excluded frameworks that were sector-specific, incomplete or unavailable online. We identified 12 frameworks that specifically focus on data ethics principles and values. An examination of these 12 frameworks allowed us to assess how ethics is currently envisaged across a range of data-led projects, schemes and guidelines.

### Identification and selection of relevant literature

In a third step, we reviewed an extensive body of literature about data practices, research ethics, and data ethics from diverse disciplines. Our aim was to synthesise a range of key arguments and concepts into a transversal framework that can be adopted across the disciplines. While other authors with related interests, such as Saltz and Dewar (2019), have conducted systematic reviews of literature which reveal valuable insights, we question whether a systematic approach must be understood as a ‘gold standard’ or as always appropriate to address research questions. A systematic approach can be most effective when the inclusion criteria for relevance to the topic under investigation can be defined very precisely, while still returning a significant number of results; this may not be the case where newer topics of inquiry are concerned. Furthermore, in order to exclude ‘lower-quality’ sources, systematic reviews tend to depend on proprietary academic databases which favour highly-cited journals and therefore typically reflect the perspectives and research outcomes of authors based in the Global North and better-resourced, research-intensive universities, reinforcing existing knowledge inequalities (Almeida & Goulart, 2017; Kordzadeh & Ghasemaghaei, 2022). In our study, we resist the notion that the purpose of conducting a literature review is always to systematically identify ‘what is already known’ about the topic, as knowledge claims arise from a wide range of highly unequal stakeholders with different interests.

Identifying relevant literature within this context requires both searching with a wide net and reviewing from a critical perspective. To this end, we adopted an approach known as critical interpretative analysis, a type of non-systematic review that aims to gather the key ideas within a field (Dixon-Woods et al, 2006; McDougall, 2015). In our case, the primary purpose of our literature review was to identify and draw together key and critical perspectives on aspects of an emerging field; therefore, our approach to selection of sources favoured thematic salience. In order to scope the size of the potentially relevant literature we conducted searches using Google Scholar. While we acknowledge Google Scholar itself is not free from algorithmic biases, studies have found that it indexes a wider number of journals as well as other sources and research outcomes than widely-used proprietary scholarly databases, thereby surfacing reports and other types of grey literature (Martín-Martín et al., 2018), which contain essential criticism and ideas which help to broaden the inclusion of underrepresented voices in our review. We started our search by using the search string (“data ethics” OR “digital ethics” AND “education” OR “course” OR “classes” OR “teaching”), and specified in the review process the search to include additional sources of promising research areas.

We excluded sources that did not meet our broad thematic requirements. Thus, we tended to exclude articles which focused on sector or discipline-specific areas of knowledge (e.g. health, engineering, computer science) as these generally focus on the application of frequently discussed concepts in a given context, and also, literature reviews that did not reveal new significant findings in the field (Hammersley, 2020; Powell et al., 2022). When assessing quality of the sources, we kept in mind that grey literature (such as reports by international and independent organisations) may not strictly meet the highest standards of academic rigour (e.g. anonymous peer review), but nonetheless contain essential criticism and ideas. We therefore opted to judge quality on a case by case basis, instead of using blanket criteria, to incorporate a wide range of underrepresented voices in our review.

### Thematic analysis and collection of ethical principles

Thematic analysis was performed following Braun and Clarke (2006) in order to distil a set of emerging key issues and topics for further attention. The preparation phase consisted of collecting suitable data for content analysis, organising the data, and selecting the unit of analysis, which in this case were the discussion of data ethics in course descriptions, data ethics frameworks, and academic literature. A qualitative analysis of textual data was conducted using this technique which involves the identification of core concepts through the review of the frequency of units of meaning, indicators, keywords and patterns in texts (Krippendorff, 2004). To identify the main themes in our data we performed a deductive analysis for the syllabi and an inductive analysis for the data ethics frameworks and the academic literature. As the syllabi mainly mentioned well-known terms to attract learners, we made a deductive analysis. In contrast, for frameworks and academic literature, an inductive analysis was chosen as there is no consensus on the main categories or principles under discussion, and because the identification of underrepresented ideas and emerging themes was part of our research aims (Elo & Kyngäs, 2008; Vaismoradi et al, 2013). To reduce biases, the themes identified by one author were reviewed and discussed by the other two authors until consensus was reached (Elo et al, 2014). Here we made use of our expertise and background knowledge as a research team coming from different disciplines: library and information science, media and communication studies, education and pedagogy, and applied philosophy.

As a last step, to develop the ethical framework we collected from all three sources action-guiding ethical principles. We subsumed closely related ethical principles, such as “do not harm” and “non-maleficence”, or “fairness” and “justice”, in a single principle by using the most-commonly referred to term. In cases of doubt, we discussed this synthesis between the authors until consensus was reached.

## Results

In our sources, data ethics was mostly understood as the process undertaken to ensure responsible and sustainable data practices across its whole cycle, from collection and management to analysis and publication, to understand the uses and risks of present and future uses of data. We observed that despite a broad consensus on the need for an ethical lens when examining the potential uses of data, the different sources placed a different emphasis on how and why we should address ethical issues.

### Analysis of research methods and data science syllabi

To understand how ethics and data ethics were taught in research methods and data science courses, we reviewed the details of the curricula including the reading lists to identify inclusions of bibliographic references to data ethics, critical approaches to the use of data, or related elements such as general ethics, ethics of research or research data management. Hereby we observed that quantitative courses tend to lack scholarly and practical literature on these topics. In the case of data science syllabi, the core focus is the technical skills needed to work with data, which reflect industry practice and priorities. The geographic distribution of the syllabi reviewed and levels at which courses were taught are presented in Table 1.

**Table 1:** Geographic distribution of the sample

		Level			
		Undergraduate	Masters	Doctoral	Data science
Country	Brazil	15	8	6	9
	United Kingdom	23	15	6	18
	United States	24	10	8	14
	Italy	6	9	8	7
	Portugal	6	6	4	6
	Spain	15	12	6	6
	France	12	9	6	8
	Germany	17	12	7	12
	Total	118	81	51	80

The distribution of data ethics elements can be seen in Table 2.

**Table 2:** Analysis of research methodology courses

Analysis of research methodology courses			
Course units	UG	Masters	PhD
Ethics of research and informed consent	42/118	58/81	24/51
Data ethics	12/118	27/81	3/51

Type of course	Quant	Qual	
Ethics of research and informed consent	65/114	59/136	
Data Ethics	27/114	15/136	

Type of course	Include ethics readings	
Quantitative	54/114	
Qualitative	97/136	

Data Science programmes	
Course units	Number of course units
Ethics of research and informed consent	12/80
Data ethics	25/80

In general terms, we did not find significant differences between the variables, but it is relevant to report that overall 52% have a unit on research ethics, which tend to focus in particular on the need to gain informed consent from participants, including the provision of detailed information about the meaning of their participation and what will be done with the information collected. However, only 16.8% go deeper by addressing issues related to data ethics, including ethical norms on data creation, collection, management, analysis, preservation, and more complex legal issues, such as privacy and data protection.

In research methods courses most of data ethics is taught in quantitative courses. However, the number of bibliographic references on data ethics is slightly higher in qualitative than in quantitative courses. Yet, at the same time, research methods courses are lacking pedagogic approaches to develop a thorough understanding of data ethics across the whole research process, from research design to science communication, that go beyond issues of anonymity. The ethics elements of these courses tend to focus on research ethics and integrity, critical appraisal of data, and analysis and interpretation of quantitative and qualitative data. Most of the reviewed syllabi structure research methods courses in introductions to research; development of hypothesis; conceptualising and conducting a research proposal; literature reviews; quantitative and qualitative research designs, methods, instruments; data analysis and presentation, and the importance of research ethics including research integrity (highlighting data fabrication and falsification). Some syllabi include elements of ethnographic observation; exploratory interviewing and focus groups; theory of science; lab design of experiments; field and natural experiments and principles and techniques of statistical analysis. Overall, there is limited presence of topics such as evaluation of ethical dilemmas, data collection, sampling, and power asymmetries and issues such as privacy.

The data science programmes tend to give only limited attention to personal data agency (consent, privacy), or the socio-technical relationships between data and power (justice, sovereignty), that is, the relations of individuals as members of specific communities with today’s ubiquitous data-intensive technologies. Furthermore, we noticed little information on ethical issues derived from uses of data, anonymisation, storage, distribution, management, reuse and publication of results, as teaching emphasises on legislation and legal frameworks instead of the ethical implication of data misuses. We observed that the data science curricula have a strong technocentric nature at the cost of humanistic approaches to data and data-driven technologies. There seems to be little discussion of how to involve participants and communities in research design, the challenges of fair recruitment and inclusion of vulnerable and subordinate people, while at the same time considering their well-being and anonymity.

Both types of curricula are missing opportunities to enhance the skills of learners in research ethics beyond informed consent and privacy, as there is little discussion on the syllabi and reading lists on issues related with other principles of data ethics, responsible uses of data, data power dynamics, data biases, data justice, data feminism, and indigenous data sovereignty, or other approaches which support learners and educators to situate research in the range of emerging ethical issues related with the datafication of society.

### Analysis of the data ethics frameworks

In the second stage of our analysis, we reviewed 12 data ethics frameworks from academic, private and civil society sectors. We list the overarching ethical principles within these frameworks in Table 3.

**Table 3:** Overview of data ethics frameworks

Name	Sector	Source	Overarching Principles
Data Feminism	Academia	D’Ignazio and Klein (2020)	• Examine power • Challenge power • Elevate emotion and embodiment • Rethink binaries and hierarchies • Embraces pluralism • Consider context • Make labour visible
Data Ethics Principles	Academia & Civil Society	DataEthics.eu (2017)	• The human being at the centre • Individual data control • Transparency • Accountability • Equality
CARE Principles for Indigenous Data Governance	Academia & Civil Society	Global Indigenous Data Alliance (2019)	• Collective benefit: for inclusive development and innovation, improved governance and citizen engagement, and equitable outcomes • Authority to control: recognizing rights and interests, data for governance, and governance of data • Responsibility: for positive relationships, expanding capability and capacity, and indigenous language and worldviews • Ethics: for minimising harm and maximising benefit, justice, and future use
Global Data Ethics Pledge	Civil Society	Data for Democracy (2021)	• Fairness • Openness • Reliability • Trust • Social Benefit
Australia’s AI Ethics Principles	Government	Australian Government, Department of Industry, Science, Energy and Resources (2019)	• Human, social and environmental wellbeing • Human-centred values • Fairness • Privacy protection and security • Reliability and safety • Transparency and explainability • Contestability • Accountability
General standards for data governance	Government	Datenethik-kommission (2019) [Germany]	• Background principles: Human dignity, self-determination, privacy, security, democracy, justice and solidarity, and sustainability • Foresighted responsibility • Respect for the rights of the parties involved • Data use and data sharing for the public good • Fit-for-purpose data quality • Risk-adequate level of information security • Interest-oriented transparency
An Ethics Framework for the Data and Intelligence Network*	Government	Scottish Government (2021)	• Responsible • Accountable • Insightful • Necessary • Beneficial • Observant • Widely Participatory
Data Ethics Framework	Government	UK Government (2020)	• Background principles: transparency, accountability and fairness • Define and understand public benefit and user need • Involve diverse expertise • Comply with the law • Review the quality and limitations of the data • Evaluate and consider wider policy implications
Federal Data Strategy Data Ethics Framework	Government	US Government (2019)	• Uphold applicable statutes, regulations, professional practices, and ethical standards • Respect the public, individuals, and communities • Respect privacy and confidentiality • Act with honesty, integrity, and humility • Hold oneself and others accountable • Promote transparency • Stay informed of developments in the fields of data management and data science
Good Practice Principles for Data Ethics in the Public Sector	International Organisation	OECD (2021)	• Manage data with integrity • Be aware of and observe relevant government-wide arrangements for trustworthy data access, sharing and use • Incorporate data ethical considerations into governmental, organisational and public sector decision-making processes • Monitor and retain control over data inputs, in particular those used to inform the development and training of AI systems, and adopt a risk-based approach to the automation of decisions • Be specific about the purpose of data use, especially in the case of personal data • Define boundaries for data access, sharing and use • Be clear, inclusive and open • Publish open data and source code • Broaden individuals’ and collectives’ control over their data • Be accountable and proactive in managing risks
Our Principles (SAS)	Private sector—Tech Company	SAS Analytics (2022)	• Human Centricity: Promote human well-being, human agency and equity • Inclusivity: Ensure accessibility and include diverse perspectives and experiences • Accountability: Proactively identify and mitigate adverse impacts • Transparency: Openly communicate intended use, potential risks and how decisions are made • Robustness: Operate reliably and safely, while enabling mechanisms that assess and manage potential risks throughout a system’s life cycle • Privacy & Security: Protect the use and application of an individual's data
Universal principles of data ethics**	Private sector—Tech Company	Accenture (2016)	• The highest priority is to respect the persons behind the data • Attend the downstream uses of datasets • Provenance of the data and analytical tools shapes the consequences of their use • Strive to match privacy and security safeguards with privacy and security expectations • Always follow the law, but understand that the law is often a minimum bar • Be wary of collecting data just for the sake of more data • Data can be a tool of inclusion and exclusion • As much as possible, explain methods for analysis and marketing to data disclosers

*The data ethics framework was developed in relation to the COVID-19 pandemic

**Accenture has issued a new revised guideline that combines data and AI ethics

The most frequently found principles are respect for autonomy (in terms of consent) and privacy, which are mostly discussed in the context of big-data and machine-learning, raising awareness of the need to inform users of how their data is being collected, processed and protected. Another frequently mentioned concern relates to data governance, explained as the set of rules that should apply to all to regulate data-led activities and to put people before data, which is understood as using data to solve social problems and to improve the quality of life.

The frameworks developed in academic contexts emphasise that human beings have to be placed at the centre of data led projects, and encourage examining or even challenging the power dynamics embedded in data ecosystems, while promoting transparency and responsible uses of data. Government-led frameworks have strong human-centred values, and tend to have a quite important legal dimension, aiming at supporting organisations to define and understand public benefits in data projects to ensure these act with honesty, integrity, and humility, respecting the public, individuals, and communities. The other key principles are transparency and accountability, condemning loopholes and impunity. The civil society frameworks suggest organisations to guarantee the security of data, individuals, and algorithms to prevent unauthorised uses of data, and to acknowledge and mitigate unfair bias throughout all aspects of data work. Likewise, frameworks developed in the private sector encourage organisations to give the highest priority to the persons behind the data and to adhere to data governance and AI model governance to advance proper AI ethics while advocating for ensuring privacy, accountability and legal compliance, while promoting the idea of self-regulation following internal codes of good practice.

The common elements of these frameworks are having a human-centred approach to uses of data, ensuring that individuals and organisations comply with the law, and making use of data governance protocols to ensure privacy and prevent biases. Although seldom stated explicitly, we can observe a broad concern that irresponsible practices and misuse will lead to tighter regulations and hesitancy to share data, limiting the freedom to work with data and increasing administrative burdens.

### Literature review

In order to look beyond the conceptualisation of ethics covered by existing frameworks and incorporate critical perspectives, we reviewed a wide range of academic literature on data ethics, including critical approaches to data and critical data literacy. We found a general consensus on the need to equip students with critical data and ethics literacies to prepare them to understand diverse phenomena such as Artificial Intelligence (AI), algorithmic discrimination through automated driven decisions, digital poverty, surveillance capitalism or platform governance, and the impact of interactions with data-driven systems on themselves and others, so people can assess, anticipate, and respond to social issues that are related to the collection, processing and use of data (Al-Nuaimi, 2020; Buckingham & Crick, 2016; Kumar et al, 2020; Powell, 2018; Sloane, 2019; Wheeler, 2018).

Despite the wide range of emerging issues, not all topics received equal consideration. Our literature review allowed us to identify a number of recurrent themes that are important for data ethics:

#### Socioeconomic discrimination

Algorithmic decision making tend to adversely affect those coming from lower-income households and neighbourhoods. This kind of behaviour is discussed as automating poverty or automating inequality, where AI is used to categorise groups, or to assign or remove services such as unemployment benefits, child support, housing and food subsidies, imposing systemic oppression (Bhaumik et al., 2006; Davies, 2020; Eubanks, 2018; Kleinberg et al, 2018; Sandvig et al, 2014). Thus, it is important to protect the rights of vulnerable persons, and be vigilant to the ways in which they may be impacted by automated decisions which increasingly determine, showcase, predict and map poverty, and depicting groups in a negative way, depending on the school they attend or where they live (Atenas & Havemann, 2019; Goldkind et al., 2021; Lo Piano, 2020; UNICEF, 2019, 2020).

#### Racism

The opacity of algorithms creates black boxes, and one of the key arguments towards the need to have regulatory frameworks is the problem of the “Racist Robots”, that for example are leading to consumer lending discrimination, or preventing certain groups obtaining visas to visit or live in countries. Moreover, they can harm marginalised groups, for example, through racially profiling predictive policing which then leads to longer incarceration (Alaieri & Vellino, 2016; Bartlett et al, 2019; Brantingham, 2017; Chander, 2017; Hepworth & Church, 2018; Khalifa et al, 2014; Kuzey et al., 2019; Roth, 2010; UNESCO, 2019).

#### Sex, gender and sexuality

Women, gendered and sexual minorities can be adversely affected by algorithmic decision-making in every aspect of their lives, including access to health, services and the labour market, for example, in clinical decisions, or psychometric tests. Thus, ethical use of data must consider the experiences and needs of women and members of LGBT+ communities (Asplund et al, 2020; Beaman et al, 2009; Cirillo et al, 2020; Kleinberg et al, 2018; Lambrecht & Tucker, 2019; Ruberg & Ruelos, 2020; Zou & Schiebinger, 2018). Furthermore, there needs to be greater awareness of the biases against female researchers and even against the very research that identifies such biases as bad scientific practices or injustices, and what one can do about it (Cislak et al., 2018; Orgeira-Crespo et al, 2021).

#### Surveillance

Businesses, employers, educational organisations and governments are engaging in surveillance capitalism (Zuboff, 2015), that is the ownership of rights for secondary use of data for profit-making, to monitor our behaviour online, in shops, at work, and while studying and taking exams. Personal data is continuously captured and tracked via engagements with near-ubiquitous technology giants; a growing number of states are deploying advanced AI tools to monitor, track, and surveil citizens to accomplish a range of murky policy objectives (Andrejevic & Selwyn, 2020; Azoulay, 2019; Feldstein, 2019; Introna & Wood, 2004; Newlands, 2021).

#### Political manipulation

Closely connected to surveillance capitalism, AI has been used to target, influence and manipulate voters through social media, acting to further polarise political opinions and fuel anger and paranoia. Personal data and algorithmic social media vectors deliver targeted propaganda messages which for many, are the main source of ‘news’ about the political sphere. Consequently, radicalisation and conspiracy-theorising have become widely normalised, threatening democratic processes, indicating a need for better regulatory frameworks (Badawy et al., 2019; Bolsover & Howard, 2019; Crain & Nadler, 2019; Hood & Margetts, 2007; Véliz, 2020; Woolley & Howard, 2016).

#### Privacy

There are elements of life people want to keep as part of our private sphere and others which people are willing to make public to facilitate social life and maintain public institutions (Rabotnikof, 2005). As data in the digital age can be easily accessed over large distances in time and space, it has become a central concern for individuals and democracies (Gstrein & Beaulieu, 2022). As it is difficult to assess what consequences data sharing has, it has led to calls to minimise the amount of data collected (Véliz, 2020) and to establish codes of conduct in regard to what data can be shared, with whom and under what specific contexts, in terms of contextual integrity (Nissenbaum, 2004; Zimmer, 2018). Furthermore, search engines make necessary new methods for controlling which parts of life one wants to share and with whom, and design features that facilitate control over one’s privacy, as scholarship on boundary regulation emphasises (McDonald & Forte, 2020).

#### Data intersectionalities

Emerging from black feminist critique, the theory of intersectionality notes that each person’s identities are multiple, and therefore facilitates analysis of how people can be differentially affected by multiple layers of discrimination (Crenshaw, 1989). In a data context, data infrastructures are being introduced to predict socioeconomic behaviours, via collection and cross referencing of data points including socio-economic status, race, gender, and neighbourhood. Such processing of data aims to predict how likely certain students are to fail or succeed at school, or how much a person must pay for their car insurance, but worse, it is used in police work, to profile and predict the future criminality of members of marginalised groups. These issues have been taken up by D’Ignazio and Klein (2020) in designing their data feminism framework, which aims to embed principles of feminist theory and equity into data-related projects, as the less we understand how such systems work, the more likely it is that historically disadvantaged groups will continue to suffer from automated negative biases (McDonald & Pan, 2020).

#### Digital ecosystems

Certain technical, social and political conditions may vastly expand the possible uses and impact of data. A holistic analysis needs to go beyond concentrating on the novelty of data-intensive technologies, and study the relations in which the different entities using data stand and what these can actually do with data under the conditions and circumstances they interact (Stahl, 2021). This perspective allows us to identify data practices and data uses that strive and become dominant.

#### Levelling the field

The emergence of more powerful technologies capable to process increasingly more data to gain knowledge about human activities, are generating social asymmetries between those who own the tools, the skills, and the computational power and the subjects whose data are subject to these applications (Belbis & Fumega, 2019; Zwitter, 2014). Thus, data-led research projects must refer to and adhere to the principles and values in which human rights and personal data protection laws are based, to ensure that the uses of data do not compromise or further harm vulnerable and marginalised groups (Azoulay, 2019; Bogroff & Guegan, 2019; Kleinberg et al., 2018; Lo Piano, 2020; Sandvig et al, 2014; Zuboff, 2015).

#### Developing guiding principles

A data ethics framework must be guided by a series of propositions and guidelines to which any data research-led project or activity must adhere. This will lead to actively design fair and less biased research and motivate students to learn, from the very beginning, the value of data protection and data agency. For this, raising awareness of the role of an ethical common ground when conducting research with data considering elements such as empathy, social justice and social good will be critical (Chang & Gray, 2013; Eisen & Parker, 2004; Stockley & Balkwill, 2013; Strohmetz & Skleder, 1992).

#### Recognising the diversity of values

Data literacy programmes should be supported by a range of social values to address the diversity in a pluralistic society. The early literature on value-sensitive design has identified privacy, non-discrimination, autonomy, and safety as widely shared values concerning information technologies. Yet, at the same time Friedman et al. (2008) note that they may conflict in practice and need to be balanced. For instance, monitoring non-discrimination often requires some invasion of privacy. To identify the different values, their relationship to each other and their importance, we need to build an ethical framework that is tailored to the needs of a datafied society.

Interest in data ethics is increasing rapidly and we can see a strong diversification of themes and ethical approaches. Scandals from industry and politics have triggered strong research interest in the field, enriching the discussion with new case studies and identifying new types of threats to individuals, communities and democracy. This research area also shows a deep interaction between academic scholarship and activism, with academics becoming activists, collaborations between activists and academics, and activists effectively using academic scholarship to back their arguments.

### Identified action-guiding principles

In the analysed syllabi, ethical frameworks and scholarly literature we found repeated reference to action-guiding principles. We have synthesised the large number of ethical demands and appeals into eight action-guiding principles that constitute our extended data ethics framework (Table 4).

**Table 4:** Framework for data ethics for data literacy (built on Atenas, Havemann, Timmermann & Kuhn, 2021)

Action guiding principles	Definition	In scholarly literature	In frameworks
Respect autonomy	This aims at enabling people, primarily as individuals, to make informed decisions about the potential uses of their data, through the concept of informed consent and transparency. In this case, informed consent needs to go beyond data collections, but clearly describe how and by whom data will be used and how the results will be published	Al-Nuaimi (2020), Buckingham and Crick (2016), Powell (2018), Wheeler (2018), Sloane (2019), Kumar et al (2020), Véliz (2019)	Data feminism CARE Principles for Indigenous Data Governance Global Data Ethics Pledge General standards for data governance Federal Data Strategy Data Ethics Framework Data Ethics Principles Good Practice Principles for Data Ethics in the Public Sector Universal principles of data ethics Our Principles (SAS)
Respect privacy	To consider that some issues should not be part of the public sphere or a matter of public concern, people have a right to withhold such type of data unless there is a commonly agreed overriding reason for publicity (e.g. additional income by politicians)	Richards and King (2014), Yao-Huai (2005)., Pollach (2005), Schwartz (2011), Herschel and Miori (2017), Zimmer (2010), Lundberg et al. (2019), Stahl and Wright (2018), Véliz (2020)	Global Data Ethics Pledge Australia’s AI Ethics Principles General standards for data governance Federal Data Strategy Universal principles of data ethics Our Principles (SAS)
Promote fairness	This asks to treat like cases alike, and recognises that we may have to make special arrangements so that no one ends up undeservingly disadvantaged Researchers must assess and decide whether those being directly or indirectly involved or affected in or by the research will be endangered, exposed, put at risk, unjustly treated, profiled or classified in a derogatory manner regardless of the interests or intentions of the researchers	Jo and Gebru (2020), Stoyanovich et al. (2018), Hoffmann et al. (2018), Richterich (2018), Ienca et al. (2018), Hand (2018), Bertino et al. (2019), Jobin et al. (2019), Johnson (2014)	Data feminism CARE Principles for Indigenous Data Governance Global Data Ethics Pledge Australia’s AI Ethics Principles Data Ethics Framework
Address equality	This means that rules should apply to all unless there is a publicly acceptable reason for exemption. It refers to the legal concept of equality, which means that every person has the same rights and should be treated in the same way regardless of their personal characteristics	Tusinski Berg (2018), Bogroff and Guegan(2019), Bezuidenhout et al. (2020), Kazim and Koshiyama (2019), Puaschunder (2019), Corple and Linabary (2020), Johnson (2014)	Data Ethics Principles CARE Principles for Indigenous Data Governance As compliance and accountability for all: Data Ethics Principles General standards for data governance An Ethics Framework for the Data and Intelligence Network Good Practice Principles for Data Ethics in the Public Sector Data ethics framework Universal principles of data ethics Australia’s AI Ethics Principles Our Principles (SAS)
Do no harm	This is often also labelled as non-maleficence, and refers to preventing data uses with negative consequence, for instance, by directly exposing or allowing the identification of individuals and groups	Raymond (2017), Kitto and Knight (2019), Loukides, Mason & Patil (2018), Berman and Albright (2017), Taylor et al. (2016)	CARE Principles for Indigenous Data Governance Global Data Ethics Pledge General standards for data governance Data Ethics Framework Universal principles of data ethics Australia’s AI Ethics Principles Our Principles (SAS)
Promote sovereignty	This means that subjects, both at individual and collective level, should be in a position to decide when and what data they wish to disclose and to whom, and that a refusal to share data, should not impede or obstruct their access to key information or to participate in political, cultural, scientific and economic life, and to welfare, education and health services. It also recognizes that affected people are the best advocates for promoting their own interests without misrepresentation	Kukutai and Taylor (2016), Walter and Suina (2019), Kukutai et al. (2020), Snipp (2016), Lovett et al. (2019), Ai-min and Jia (2015), Hummel et al. (2018)	CARE Principles for Indigenous Data Governance
Address bias	Epistemic structures influence the way we think about certain people and objects, sometimes giving them undeserved advantages or disadvantages It refers to avoid portraying individuals or groups through prejudiced or predetermined ideas to influence decisions in a certain direction. Many claim that we all have biases, and that there are in a certain degree unavoidable, so we need to take action	Richterich (2018), Henderson (2019), Herschel and Miori (2017), Ienca et al. (2018), Mittelstadt et al. (2016), Buenadicha et al. (2019) McDonald and Pan (2020)	Data feminism Global Data Ethics Pledge General standards for data governance An Ethics Framework for the Data and Intelligence Network
Challenge power structures	Support individuals to confront and challenge existing power structures that exist to limit who can decide, and for how long their decision stands, and who can be forced to comply with those decisions within society, government and communities	Taylor (2016), Heeks and Shekhar (2019), Atenas et al. (2020), Dencik et al. (2016) Dencik and Sanchez-Monedero (2022) Goldkind et al. (2021)	Data feminism CARE Principles for Indigenous Data Governance As balancing power: General standards for data governance

The syllabi mostly mention two principles: privacy and respect for autonomy, usually limited to issues of informed consent.

Depending on how broad “action-guiding principles” are understood, there was another element that could be included in this framework. As research methods and data science syllabi, and ethical framework were strongly user-centric, there was repeated reference to “research integrity”. This was mainly understood in a narrow sense, as a condemning data fabrication, data manipulation and data falsification. Due to the negative consequences of these acts on science and ultimately society, teaching materials and ethical frameworks repeatedly made references to scientific integrity, professional responsibility and abiding to professional rules of conduct. Academic literature however already deals with issues of data fabrication, manipulation and falsification under principles of “promote fairness”, “no harm” and “address bias”.

Lastly, it was interesting to observe that syllabi, ethical frameworks and academic literature tended to concentrate on a smaller set of principles (or even a single principle), or place emphasis on different principles in separate publications. Our results show the main emphases within the cited documents.

## Discussion: an ethics as methods framework for critical data literacy and research methods

To address these wide range of themes and aims in the context of research-based learning activities and research methods courses, we consider that a critical approach to the ethical values concerning how we interrogate issues related to data is needed. An approach to expanding educators and learners capacities to identify and analyse ethical issues is to adopt an understanding of “ethics as methods”, as proposed by Markham et al. (2018), who note that: “Although ethics is often considered a philosophical stance that precedes and grounds action, it is a value-rationality that is actually produced, reinforced, or resisted through practice. Very quickly, indeed immediately, ethics, when practiced, becomes a matter of method.” (p. 2). In other words, learning how to “do” ethics is required if students are to identify ethical issues, analyse them and propose solutions in line with ethical norms.

A widely used approach to teach and learn ethical reasoning is the acquisition of ethical principles (Beauchamp & Childress, 2019; DeGrazia & Millum, 2021). Ethics training can start by acquiring a very basic set of principles that can be expanded. In follow-up courses and self-learning these principles can be analysed in more depth and expanded in number (see Table 4), and the relationship between these principles can be examined in constellations of increasing complexity. In this sense, data ethics can be framed within the idea of critical data literacy. When students associate a concept to a series of ethical considerations they start to think about such concepts as action-guiding principles, and they can put ethics into practice across the whole research data cycle, from the development of tools for data collection, gathering data from different sources and groups, managing and safeguarding data, analysing data and communicating their findings using an ethical approach to data storytelling and scientific communication.

Thus, for instance, by starting a discussion with a widely known concept, such as privacy, ethics teachers can motivate students to think about the many possible ethical issues that can be associated with the concept. Privacy as a principle is associated with a specific idea on how such a good is to be treated (Véliz, 2019). When other principles are added to discussion, such as respect for autonomy, students can develop on the basis of concrete examples how the principles interact. For instance, older adults are often very open to accept the privacy intrusive nature of smart sensors that monitor their movements, and can alert emergency services in cases of falls, as they are keen to regain autonomy (Predel et al, 2022). In contrast, public figures and activists have a strong interest to keep a high level of privacy, even at the cost of losing some of their autonomy, to reduce possible harms.

As we saw in Table 4, there is no definitive understanding or agreement on a fixed set of principles that should govern data ethics; rather, various authors and organisations are engaged in a struggle to set the agenda and expand or delimit the boundaries of the ethical analysis. Much of ethics training needs to be adapted to the needs and context of the learners and the society(-ies) they live and work in. Our analysis of syllabi, data ethics frameworks and academic literature has nonetheless shown that there are a few key issues that require special attention in HE: power structures, vulnerabilities and relationships, and social responsibility.

When it comes to power structures, a significant concern is that ultimately, dominant organisations are more capable of defining what ethical practices comprise (van Maanen, 2022; Washington & Kuo, 2020). It therefore becomes imperative that students learn at an early stage about the different interest groups that exert influence in data ethics discussions, so that they can identify potential conflicts of interest. Educational programmes need to build capacities in conducting research through a critical and ethical framework to enable them to challenge data power structures, by addressing structural social problems through an interdisciplinary and social justice approach (Iliadis & Russo, 2016; Dalton et al., 2016; Metcalf et al., 2016; Burns et al., 2018; BERA, 2018; Timmermann, 2018; Atenas et al., 2020; Mtawa & Nkhoma, 2020; Decuypere, 2021). Hence, for educators, a guiding question becomes: how can we ensure that training in data enables students to identify and challenge power asymmetries? Familiarity with the principle of “challenging power structures” is a first step in thinking about the ethical dimensions of power and power abuses related to data harvesting and use.

Teaching ethical research practices requires activities that promote respect for autonomy, privacy and dignity of individuals, groups and communities and how to equally distribute the risks and benefits of the research. It also requires developing a sensitivity to intersectional considerations that negatively affect vulnerable groups (McDonald & Pan, 2020) and factors that give undeserved advantages over others to those already well-off. An ethics of care perspective provides sensitivity to these issues, positioning ethics as relational, contextualised, embodied and realised through practices rather than residing in stand-alone principles. Care is considered politically, that is, in relation to the intersecting hierarchies of power and privileges that are inherent in the context of modernity. This poses further ethical challenges in terms of race, indigeneity, class and gender. A care ethics approach asks us to reflect on the question of privilege while also creating spaces to build solidarities. Using an ethical framework to enable the critical understanding of the wide spectrum of data issues in the context of HE, can therefore support educators in assessing their own teaching and research practices, and foster participatory and collaborative learning activities, co-creating knowledge for social transformation (Atenas, 2021; Atenas et al., 2021).

Conducting research with data about people is a privilege, not a right (Atenas et al., 2020; Carpenter, 2018; Dencik & Sanchez-Monedero, 2022; Floridi & Taddeo, 2016). When we conduct research with human data, we are not simply examining data points but entering into peoples lives, places and stories, their culture and beliefs. Therefore, when entering the field, we must acknowledge people and communities as subjects not objects of research. Research educational programmes must be designed to acknowledge ethical boundaries following established principles of good scientific practice and research ethics: respect for persons, beneficence and justice putting the common good at the heart of research (Carpenter, 2018; Oates, 2021). Graduates entering the commercial space, where the profit imperative may be seen to come into conflict with their ethical training, must also be equipped to make the case for ethical practice as ultimately, not only socially responsible, but better business practice. The unethical use of data will undermine the willingness to share and curate data, and expand protective legislation, thereby reducing the amount of data available for use and increasing the administrative burden to clear freedom to operate.

Lastly, ethical practice must learn to operate within a context of constant change, with the continuous development of technologies, and evolution in the rule of law and accountability of data exploiters. This requires continuously adapting ethical frameworks to emerging challenges. Under this context, it is also important that ethics training leads to assuming a certain degree of social responsibility. As Johnson (2014) and Metcalf et al. (2016) note, teaching critical data literacy involves integrating case studies with practical work, fostering collaboration, co-creation and collective responsibility by examining social privileges in data and the norms of data systems. It is important that data-led research and learning activities are designed to address inequalities, to improve quality of life, to explore issues that may be harming a community, and also to improve data governance, as it is key that people acquire the skills to participate in developing policy frameworks that go beyond data protection, and provide a fair, safe, unbiased and equitable data landscape, regulating what the public and private sectors can do with data. Data should help identify deeply-anchored inequities and emerging cases of discrimination and malpractices, and not serve as a tool to perpetuate injustices.

## Recommendations

We consider that ethics, and more specifically, data ethics in the context of teaching research methods, should be actionable. Thus, to develop critical data literacies and research skills in HE, ethics should be a transversal element rather than a formality or ‘tick-box’ exercise, hence embedding the concept of ethics as a method, guiding research from design through to communication and every stage in between (Markham, 2006; Markham et al., 2018).

In designing a curriculum for research methods and data science courses, educators should foster an understanding and discussion of ethical norms and dilemmas, and thereby, of potentially beneficial and harmful uses of data. While we have shown a clear preference for using an extended list of ethical principles in our synthesis of the different frameworks, even the use of shorter sets of principles is a substantial improvement. As most courses in use focus on the need for informed consent, thereby placing individual autonomy at the centre of the question of ethics, we strongly recommend expanding to a further set of principles to also discuss issues of social justice.

The use of ethical principles in the training of medical students has shown great success with mastering four principles: respect for autonomy, beneficence, non-maleficence and justice (Beauchamp & Childress, 2019). Based on over four decades experience of teaching ethical principles in the healthcare context, building upon the Belmont Report (National Commission for the Protection of Human Subjects of Biomedical & Behavioral Research, 1979), educators should be free to limit the set of principles for introductory courses to three or four, and expand the set of principles as course participants become more proficient in analysing the social issues of data practices. The selection of principles should depend on the ethical training of the educators, the time available and the pressing social issues course participants may encounter. The selection of ethical principles should however cover as a minimum one principle from each of the following three dimensions: (i) to defend primarily individual interests (respect for autonomy, privacy), (ii) to fight injustice (do not harm, address bias) and (iii) to promote collective well-being (promote fairness, address equality, promote sovereignty, challenge power structures). These three dimensions are based on the Belmont Report’s original emphasis of the ethical principles commonly thought in bioethics—respecting autonomy, reducing harms and promoting social justice—and have proven to be an adequate minimum standard for the ethical training of health workers which can be adapted to other professions. Learning about these three dimensions may motivate taking additional ethics courses and facilitates self-learning by being able to position additional ethical principles in a basic normative framework and identify further applications.

## Limitations

Our decision to opt for a non-systematic review and carry out a thematic analysis inductively comes at the cost of a certain bias towards our own professional interests and disciplinary perspectives. We nonetheless defend our approach as it facilitates the inclusion of voices which are often marginalised in the academic discussion or overshadowed by reports of financially strong institutions (Powell & Koelemay, 2022). Furthermore, databases that are reputable in academic venues are often dominated by for-profit publishing houses and also not free from algorithmic biases. We therefore judge that our analysis can complement previously published systematic reviews by diversifying the scope of ethical perspectives.

## Conclusions

Our purpose in this paper has been to both establish the need for, and outline, a set of action-guiding ethical principles for embedding data ethics as a core element in teaching data skills within research methods and data science training. Thus, methods and data literacy programmes should be teaching ethics beyond informed consent, as ethics itself must be considered a research method and a research praxis, making ethics actionable knowledge (Marco & Larking, 2000; Simon, 2015; Nielsen, 2016; Bonatti, et al. 2018; Decuypere, 2021). We suggest incorporating a selection or the complete set of described ethical principles in research methods courses to explore with students issues of ethics and data, including through data-led learning activities across the disciplines.

We consider, as suggested by Reijers, et al. (2018) that any research should be carried out approaching ethics as: (i) *ex ante* methods, to understand how data is used and its potential impact; (ii) *intra* methods, to understand the impact of data at different levels; and (iii) *ex post* methods, to understand how the research has had an impact in different communities. This approach can build an understanding of the core ethical elements within the discipline of study, including the codes of conduct in professional fields, with attention to the fundamental distinction between the *ethics* of data practices and *laws* which govern them, because an action may be legal but unethical, or illegal but ethical. The notion that these are the same can be an accidental or deliberate cause of confusion or obfuscation. Students therefore need to become acquainted with ethical practice both within and beyond legal and regulatory frameworks, as ethics can be understood as a method, a procedure, and a perspective to guide decisions around how to study and analyse data as social phenomena.

As scholars working on indigenous data sovereignty have repeatedly emphasised, we consider that data and research literacy should explore participatory and inclusive research design, involving those who will provide data or be affected by the research, paying attention to vulnerable communities, so that biases and prejudices that shape the presentation of results are minimised. Key to achieving this is to acknowledge that humans are not objective (Saini, 2020) and systematically incorporate diverse viewpoints. A practical way to do this is to co-construct the data with the participants of the study.

Thus, it is key to promote interdisciplinary dialogues and seek input from those who deal with sensitive topics, high-risk research situations, and/or vulnerable populations in the context of the epistemic and axiological shifts, where we are all potentially vulnerable, and all data are potentially sensitive (Tiidenberg, 2018). We consider that it is important that curriculum design is person- and community- centred, this will include supporting people in finding ways to be in control and empowered by their data, as well as challenging pervasive power dynamics and making data users accountable for their actions.

## Data Availability

No additional data is associated to this research.
